# Glycogen synthase kinase-3 beta inhibitors protectagainst the acute lung injuries resulting from acute necrotizing pancreatitis^1^

**DOI:** 10.1590/s0102-865020190060000009

**Published:** 2019-08-19

**Authors:** Hongzhong Jin, Xiaojia Yang, Kailiang Zhao, Liang Zhao, Chen Chen, Jia Yu

**Affiliations:** I PhD, Department of Hepatobiliary Surgery, Renmin Hospital, Wuhan University, Hubei Province, China. Acquisiton and analysis of data, manuscript writing.; II MD, PhD, Department of Hepatobiliary Surgery, Renmin Hospital, Wuhan University, Hubei Province, China. Conception and design of the study, supervised all phases of the study, final approval.; III MD, PhD, Department of Hepatobiliary Surgery, Renmin Hospital, Wuhan University, Hubei Province, China. Statistical analysis, manuscript preparation.; IV MD, PhD, Department of Hepatobiliary Surgery, Renmin Hospital, Wuhan University, Hubei Province, China. Technical procedures, histopathological examinations.

**Keywords:** Glycogen Synthase Kinase 3 beta, Pancreatitis, Acute Necrotizing, Acute Lung Injury, NF-kappa B, Rats

## Abstract

**Purpose:**

The research is intended for clarification of the efficacy as well as the underlying mechanism of GSK-3β inhibitors on the advancement of acute lung injuries in acute necrotizing pancreatitis (ANP) in rats.

**Methods:**

Seventy-two rats were randomly divided into 6 groups: (1)ANP-vehicle; (2)ANP-TDZD-8;(3)ANP-SB216763;(4)Sham-vehicle;(5)Sham-TDZD-8;(6)Sham-SB216763; Blood biochemical test, histopathological examination and immunohistochemical analysis of rats pancreas and lung tissues were performed. The protein expression of GSK-3β, phospho-GSK-3β (Ser9), iNOS, ICAM-1, TNF-α, and IL-10 were detected in lung tissues by Western-blot.

**Results:**

The outcomes revealed that the intervention of GSK-3β inhibitors alleviated the pathological damage of pancreas and lung (P<0.01), reduced serum amylase, lipase, hydrothorax and lung Wet-to-Dry Ratio, attenuated serum concentrations of IL-1β and IL-6 (P<0.01), inhibited the activation of NF-κB, and abated expression of iNOS, ICAM-1 and TNF-α protein, but up-regulated IL-10 expression in lung of ANP rats (P<0.01). The inflammatory response and various indicators in ANP-TDZD-8 groups were lower than those in ANP-SB216763 groups.

**Conclusions:**

Inhibition of GSK-3β weakens acute lung injury related to ANP via the inhibitory function of NF-κB signaling pathway. Different kinds of GSK-3β inhibitors have different effects to ANP acute lung injury.

## Introduction

ANP is an acute abdomen-characterized disease, and it is featured with the advancement of systemic inﬂammatory response syndrome and multiple organ failure syndrome^1^. Acute pancreatitis associated-lung injury (APALI) refers to commonly-seen and known serious complications of ANP^2^. Upon it happening, APALI could cause speedy advancement of the disease and increase mortality in ANP patients^3^. However, the precise mechanism of APALI occurring in ANP patients is still not completely understood; increasing findings show the pathological sequelae of ANP and ANP-related organ failures under the mediation of pro-inﬂammatory cytokines as well as adhesion molecules, comprising TNF-α^4^, interleukin-1β (IL-1β), interleukin-6 (IL-6) and adhesion molecules (such as ICAM-1) may cause systemic inflammation. At the same time, anti-inflammatory factor (such as IL-10) is restrained and is one of the important factors^5,6^. Hence, decreasing the harmful pro-inﬂammatory mediators to inhibit the inflammation effectiveness could be an effective treatments for APALI.

Glycogen synthase kinase-3 (GSK-3) is a universally expressed protein kinase siting in the nexus of multiple signaling pathways^7^. GSK-3’s unique characteristics mean the display of great activity in basal conditions, which can be regulated in a differential manner by tyrosine and S/T phosphorylation. Tyrosine phosphorylation (Tyr279 for GSK3α and Tyr216 for GSK3β) can make it more active, while N-terminal serine phosphorylation (Ser21 for GSK3α and Ser9 for GSK3β) is suppressive^8^. A growing number of studies have demonstrated direct or indirect inactivation of GSK-3β inhibits the innate response, and provides immune protection to the damaged organ^9-11^. Growing researches displayed that effective selective inhibitors of GSK-3β relieve organ damage or dysfunction associated with sepsis, pancreatitis, ischemia-reperfusion injury, critical illness et al^12^. Such discoveries are the basis for an assumption that GSK-3β may play a vital part in regulating the inflammation responses. Nevertheless, the effectiveness of GSK-3β inhibition in the prevention of APALI has not yet been elucidated.

GSK-3β inhibition has been assumed to be likely to restrain the inflammation of APALI. In the present study, different GSK-3β inhibitors were used: non-ATP competitive inhibitor TDZD-8 and small molecule ATP competitive inhibitor SB216763 to intervene ANP rats to observe the protective effect of GSK-3β inhibitors on APALI. At the same time, they were used to observe whether there are different effects of different types of GSK-3β inhibitors.

## Methods

All procedures used in the animal experiments conformed to the international guidelines for the care and use of laboratory animals, and were approved by the Laboratory Animal Welfare & Ethics Committee (IACUC) of Wuhan University (WDRM. NO.20171019).

### Antibodies and reagents

TDZD-8, SB216763 and sodium taurocholate were purchased from Sigma. NF-κB p65, GSK-3β, p-GSK-3β(ser9), iNOS, ICAM-1, IL-10, TNF-α and β-actin antibodies were purchased from CST Inc. (Danvers, MA, USA). Rat IL-1β, IL-6 ELISA kits were purchased from Cusabio Corp (Wuhan, China). Nuclear-Cytosol Extraction Kit were purchased from Applygen Technologies Inc (Beijing, China).

### Animals and model

Male SPF Wistar rats, weighing 200 to 250g, were bought from the center of experimental animal of Hubei Academy of Medical Sciences (Wuhan, China). Before the induction of pancreatitis, the animals were fed with standard laboratory rodent chow, and were allowed free access to sterile water. Rats were randomly divided into 6 groups (Table 1). All the rats underwent the operation and were euthanized after 12h. The dose of TDZD-8 and SB216763 used here to reduce acute lung injury was chosen based on a previous study in which similar doses of TDZD-8 and SB216763 had previously been shown to exert protective effects in experimental models of inflammatory response^13,14^. Subcutaneous infusion of sterile saline (2 ml/kg) into all rats was then performed to compensate for the estimated fluid loss.

**Table 1:** Experimental groups.

Groups	n	Models	Operations	Pretreatment
Sham-vehicle	12	Sham	saline (1ml/Kg)	10% DMSO
ANP-vehicle	12	ANP	5% sodium taurocholate (1ml/Kg)	10% DMSO
Sham-TDZD-8	12	Sham	saline (1ml/Kg)	TDZD-8 (1mg/Kg)
ANP-TDZD-8	12	ANP	5% sodium taurocholate (1ml/Kg)	TDZD-8 (1mg/Kg)
Sham-SB216763	12	Sham	saline (1ml/Kg)	SB216763 (1mg/Kg)
ANP-SB216763	12	ANP	5% sodium taurocholate (1ml/Kg)	SB216763 (1mg/Kg)

ANP: Acute necrotizing pancreatitis; Sham: Sham operation; All the ANP groups rats were established by retrograde infusing 5% sodium taurocholate (1ml/kg) into the biliopancreatic duct under standardized pressure control, and all the Sham groups rats were subjected to an equivalent volume of the saline. Pretreatment: 30 minutes before modeling, the corresponding medicines were injected through the femoral vein. 10% DMSO is the vehicle of TDZD-8 and SB216763.

### Sample collection

Blood samples were obtained from the inferior vena cava after sacrifice, followed by centrifugation for 10 min at 12.000*g*. Serum was cryopreserved at -80°C. Pancreas and lung tissues were transferred to liquid nitrogen immediately and cryopreserved at -80°C until examination.

### Histopathology analysis

Paraffin-embedded pancreatic and lung specimens were sectioned at 4μm and stained with HE. The morphological study was performed under the optical microscope by two professional knowledge investigators who were blinded to the experiment. Pancreatic histological assessment was determined by edema, hemorrhage, vacuolization, inflammatory cell infiltration, and acinar necrosis according to the standard scale system described by Schmidt *et al*.^15^. Similarly, lung injury was assessed using a scale for alveolar septal thickening, alveolar hemorrhage and inﬂammatory cell infiltration and fibrosis, as described by Werner *et al*.^16^.

### Serum enzyme activity assay

Serum specimens needed to be segregated so as to conduct biochemical and cytokine measurements. Measurement of plasma amylase (AMY) and lipase (LIPA) was performed through the use of an automatic biochemistry analyzer with standard techniques (Olympus Optical Ltd., Japan).

### Measurement of lung Wet-to-Dry ratio

The degree of pulmonary edema was determined through calculation of the wet/dry ratio from the initial weight of the right lung middle lobe (wet weight) to its weight after desiccation at 70C for 24h (dry weight).

### Measurement of hydrothorax

Weighing each dry cotton balls weight (Dry weight, g), with dry cotton balls adsorption each rat **hydrothorax** and weighing them again (Wet weight, g). Calculation formula is: hydrothorax = Wet cotton ball weight- Dry cotton ball weight.

### Measurement of cytokines

Detection of the serum concentrations for IL-6, and IL-1β was performed through enzyme-linked immunosorbent assay (ELISA) utilizing relevant ELISA kits in line with the protocol of vendor. An automated microplate reader at 450 nm was utilized for reading the absorbance; calculation of the concentrations was conducted based upon the standard curve run on each assay plate. Every specimen was repeated thrice.

### Immunohistochemistry of NF-κB p65

Detection of NF-κB p65 protein expressions was carried out in the pancreatic and lung tissue by immunohistochemistry. Pancreatic and lung tissue placed in decalcifying solution for 24 h; preparation of 4 μm sections was made out of paraffin embedded tissues. After deparaffinization, with the utilization of 0.3% hydrogen peroxide, the endogenous peroxidase activity was inactivated. Nonspecific adsorption was minimized by incubation of the section in 5% normal goat serum in phosphate-buffered saline. Endogenous biotin and avidin binding sites were blocked by avidin and biotin, respectively. Incubation of sections was carried out overnight with rabbit polyclonal anti-rat NF-κB p65 antibody: 100 in PBS, v/v in a moisture chamber. Hematoxylin was utilized to counterstain the sections. PBS was utilized rather than primary antibody when conducting negative control studies. Immuno- histochemical staining analysis was conducted with utilization of Image Pro-Plus (version 6.0). In short, the positive staining zones were selected as the area of interest (AOI), and the area sum and integrated optical density^17^ of the AOI were selected as measurement parameters. The target protein expression index is equal to the quotient between the IOD and the total AOI. Finally, statistically analysis of the average expression index of all replicates was performed.

### Western-blot analysis

Western blot analysis was utilized so as to make a determination of the expression of GSK-3β, phospho-GSK-3β (ser 9), iNOS, ICAM-1, TNF-α and IL-10 in the lung tissue. Nuclear-Cytosol Extraction Kit was also adopted to extract the proteins. The protein concentrations of the specimens were determined using the Bradford approach with bovine serum albumin as the standard. In brief, identical quantities of protein specimens were electrophoresed in 8% or 10% sodium dodecylsulphate polyacrylamide (SDS-PAGE) gels and transferred to polyvinylidene diﬂuoride (PVDF) membranes (Millipore). The membrane was blocked with TBST buffer (TBS comprising 5% fat-free dry milk, 0.1% Tween-20) at room temperature for 2 hours and then incubated with the primary antibodies (all were suggested to dilute in line with 1:1,000) and overnight at 4°C. After washing with TBST (5 min x 3), incubation of the membranes was performed utilizing fluorescence labeling secondary antibody at room temperature for 1 to 2 hours. Then Odyssey Infrared Imaging System (LI-COR Biosciences, Lincoln, NE, USA) was utilized to scan the certain protein bands instructed by its vendor. Quantity One 4.6.2 software (Bio-Rad Laboratories, Inc., Hercules, CA, USA) was also utilized for quantification of the relative band intensity.

### Statistical analysis

Statistical analyses were carried out with Microsoft Excel analysis tools or GraphPad Prism 6.0 software. All the information was indicated as means ±standard deviation (s.d.). With the adoption of student’s t-test, a comparison was performed between the two groups (*P* < 0.05 indicating statistical significance).

## Results

### Effects of GSK-3β inhibitory function on pancreatic and lung injuries in ANP

Serum amylase and lipase are thought to be the markers of acute pancreatitis with greatest sensitivity and specificity; an assessment of the activities of those markers was performed by us. ANP-TDZD-8 and ANP-SB216763 groups produced a reduction of amylase and lipase at 12 hours after modeling, versus ANP-vehicle group. Rats subjected to ANP had a growth in hydrothorax and pulmonary edema, revealing that rats were experiencing aggravated pulmonary dysfunctions. A significant improvement was seen in the ANP-induced pulmonary function alterations through TDZD-8 and SB216763 pretreatment (*P*<0.01). In addition, except for hydrothorax, the ANP-TDZD-8 showed better therapeutic effects than SB216763 (*P*<0.01). In sham-vehicle, sham-TDZD-8 and sham-SB216763 groups, there was no increase in the serum levels of amylase and lipase, hydrothorax and pulmonary edema (Table 2).

**Table 2:** Detection of pancreas and lung function indexes in each group of rats.

Groups	n	AMY(U/L)	LIPA(U/L)	Hydrothorax (g)	Lung(W/D) Ratio
Sham-vehicle	12	1560±66.11	48.3±4.59	0.20±0.04	1.52±0.06
ANP-vehicle	12	10073±343.10^a^	2375±51.14^a^	6.83±0.49^a^	3.35±0.15^a^
Sham-TDZD-8	12	1502±88.25	52.7±5.77	0.21±0.03	1.37±0.04
ANP-TDZD-8	12	4018±195.60^abc^	848.6±90.64^abc^	3.05±0.39^abc^	2.24±0.09^abc^
Sham-SB216763	12	1442±69.94	56.2±4.75	0.22±0.05	1.42±0.07
ANP-SB216763	12	5272±133.40^abde^	964.6±88.79^abde^	3.42±0.33^abe^	2.59±0.08^abde^

AMY: amylase; LIPA: lipase; Lung (W/D) Ratio: lung Wet-to-Dry ratio; ^a^P<0.01, compared with Sham-vehicle groups; ^b^P<0.01, compared with ANP-vehicle groups; ^c^P<0.01, compared with Sham-TDZD-8 groups; ^d^P<0.01, compared with ANP-TDZD-8 groups; ^e^P<0.01, compared with Sham-SB216763 groups.

### Effects of GSK-3β inhibitions on the degree of pancreatic and pulmonary histopathology

**Figure 1:**
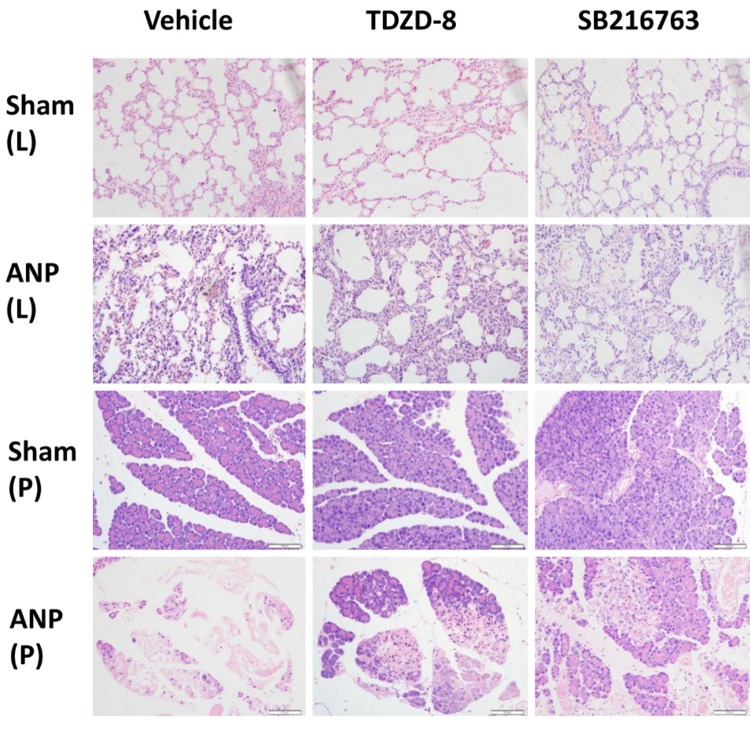
Morphologic changes in the lung and pancreas at 12 hours after sodium taurocholate induced acute necrotizing pancreatitis. No histological alterations were observed in the pancreatic and lung tissues obtained from sham-vehicle, sham-TDZD-8 and sham-SB216763 rats. TDZD-8 and SB216763 pre-treatment significantly reduced the extent and severity of the histological signs of pancreatic and lung injury. This figure represents at least 3 experiments performed on different experimental days (original magnification, ×200).

Typical histological sections are shown in Figure 1. STC-induced pancreatic injuries were featured with elevated edema, inflammatory cell infiltration, vacuolization and necrosis. Sham groups animals displayed less morphological evidence of pancreas injuries apart from mild interstitial edema.

According to Table 3, there was a significant reduction of pancreatic histological score in ANP rats which were pretreated with TDZD-8 or SB216763 (P<0.01). In addition, the pancreatic pathological score of the ANP-TDZD-8 group was lower than that of the group ANP-SB216763 (P<0.01). In Sham groups, the histological characteristics of the pancreas were typical of a normal architecture.

**Table 3:** Pancreatic and pulmonary histological score, plasma levels of IL-1β and IL-6 in each group of rats.

Groups	n	Pancreatic histological score	Pulmonary histological score	IL-1β (ng/L)	IL-6 (ng/L)
Sham-vehicle	12	0.42 ± 0.15	0.38 ± 0.15	55.7 ± 3.09	95.8 ± 1.03
ANP-vehicle	12	12.08 ± 0.30^a^	9.00 ± 0.82^a^	299.1 ± 15.46^a^	385.4 ± 13.92^a^
Sham-TDZD-8	12	0.43 ± 0.28	0.42 ± 0.15	55.4 ± 2.82	94.8 ± 0.91
ANP-TDZD-8	12	5.92± 0.39^abc^	5.37 ± 0.52^abc^	194.7 ± 12.17^abc^	180.0 ± 10.18^abc^
Sham-SB216763	12	0.50 ± 0.18	0.45 ± 0.18	51.2 ± 2.63	99.7 ± 2.03
ANP-SB216763	12	6.75 ± 0.38^abde^	7.42 ± 0.62^abde^	220.0 ± 17.70^abde^	212.6 ± 9.32^abde^

IL-1β, interleukin-1β; IL-6, interleukin-6; ^a^P<0.01, compared with Sham-vehicle groups; ^b^P<0.01, compared with ANP-vehicle groups; ^c^P<0.01, compared with Sham-TDZD-8 groups; ^d^P<0.01, compared with ANP-TDZD-8 groups; ^e^P<0.01, compared with Sham-SB216763 groups.

In comparison with Sham groups, animals undergoing pancreatitis for 12 hours showed the recognizable characteristics of lung injuries, alveolar wall thickening, and growing exudates, as well as inﬂammatory cell infiltration in the alveolar spaces (Fig. 1). Lung tissues acquired out of rats treated with TDZD-8 and SB216763 showed decreasing histological characteristics and pathological grading of lung injuries in contrast with ANP-vehicle rats (P<0.01). In addition, the pulmonary pathological score of the ANP-TDZD-8 group was lower than that of the ANP-SB216763 group (P<0.01) (Table 3).

### Effects of GSK-3β inhibitory function on IL-6 and IL-1β production after ANP

An analysis of the plasma level was performed for testing whether GSK-3β inhibitory function can modulate the inﬂammatory procedure via the regulation of IL-1β and IL-6. From Table 3, IL-1β and IL-6 had a sharp elevation in plasma of the ANP-vehicle group. In contrast, a significant reduction was seen in plasma levels of IL-1β and IL-6 among animals that had received TDZD-8 or SB216763 (P<0.01). In addition, the plasma levels of IL-1β and IL-6 in ANP-TDZD-8 group rats are lower than that of the ANP-SB216763 group (P<0.01). The plasma levels of IL-1β and IL-6 were not elevated from sham-vehicle, sham-TDZD-8 and sham-SB216763 groups animals (Table 3).

### Effects of GSK-3β inhibitors on the expression of NF-κB p65 in pancreas and lung tissues after ANP

**Figure 2:**
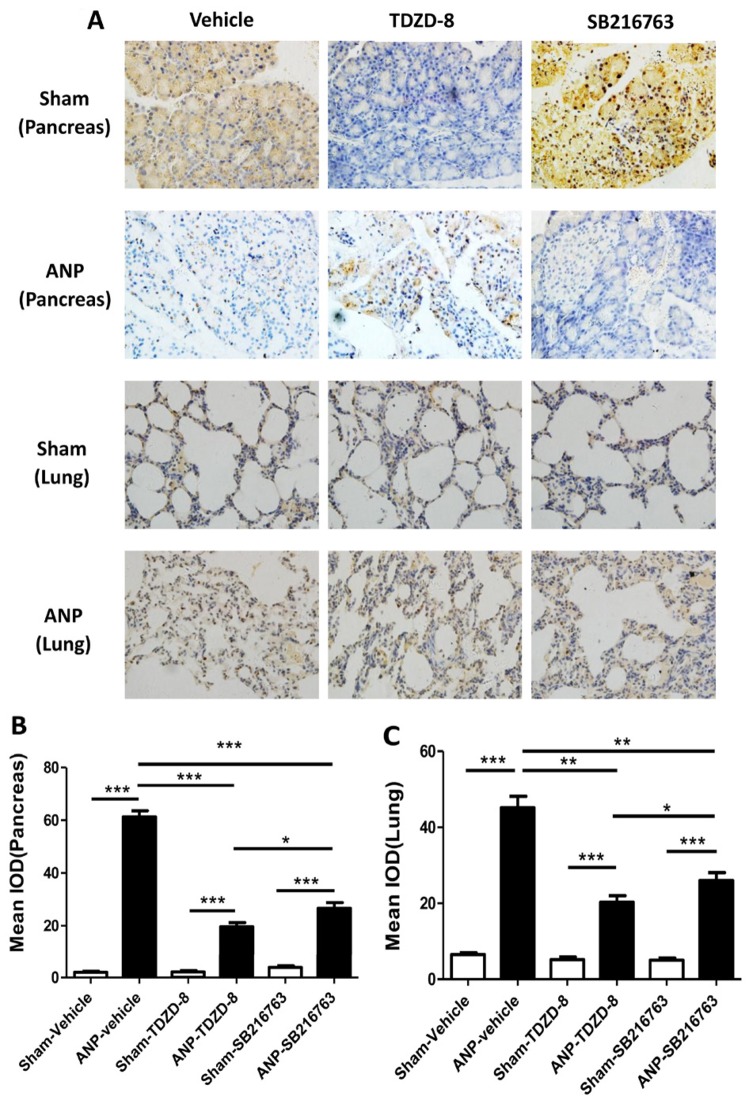
Immunohistochemical localization of NF-κB p65 in the pancreas and lung following ANP. A. Weak immunoreactivity in the cytoplasm was observed in pancreatic and lung sections obtained from sham-vehicle, sham-TDZD-8 and sham-SB216763 rats. In contrast, intense positive staining in the nucleus was observed in the pancreatic and lung sections obtained from ANP-vehicle group rats. The intensity of the positive staining in the nucleus for NF-κB p65 was markedly reduced after the administration of TDZD-8 or SB216763. But the expression of NF-κB p65 in the ANP-SB216763 group was slightly stronger than that in the ANP-TDZD-8 group. The figures represent at least three experiments performed on different experimental days (original magnification ×400). B. Immunohistochemical analysis of NF-κB p65 in pancreatic tissue. Results are presented as the mean ± standard deviation. C. Immunohistochemical analysis of NF-κB p65 in lung tissue. Results are presented as the mean ± standard deviation. Statistical significance was determined using Student’s two-tailed t-test. *P < 0.05, **P < 0.01, ***P < 0.001. ****P < 0.0001. (IOD, integrated optical density.)

In performing the localization of NF-κB p65 subunit expression, immunohistochemical assay was used. As shown in Figure 2, NF-κB p65 expression was primarily expressed in the cytoplasm in pancreatic or lung tissues. Immunostaining of NF-κB p65 was obviously expressed in the nucleus, whether in the pancreatic or lung tissue, as compared to that observed in sham-vehicle animals. Compared with ANP-vehicle groups, signiﬁcant reduction was seen in the positivity of immunostaining of NF-κB p65 in rats that had received TDZD-8 or SB216763 (P<0.01). In addition, the expression of NF-κB p65 in ANP-SB216763 group was slightly stronger than that in the ANP-TDZD-8 group (P<0.01). Weak immunoreactivity mainly in the cytoplasm was observed in sham-vehicle, sham-TDZD-8 and sham-SB216763 groups rats (Fig. 2).

### Effects of GSK-3β inhibitors on the p-GSK-3β (ser9) in lung tissues after ANP

**Figure 3:**
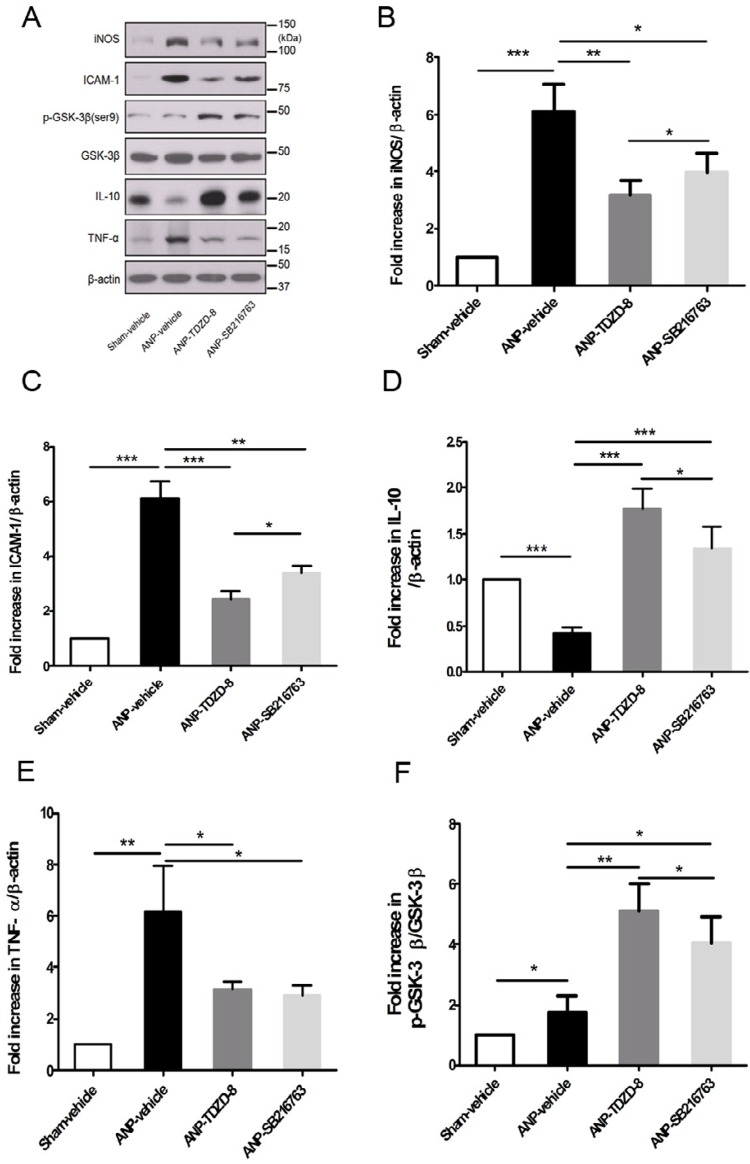
A. The activation of phospho-GSK-3β (Ser9), GSK-3β, iNOS, ICAM-1, IL-10 and TNF-α in lung tissue was analyzed by western blot at 12 hours following ANP. ANP markedly increased the tissue concentration of iNOS, ICAM-1 and TNF-α compared with the sham-vehicle rats. TDZD-8 or SB216763 pretreatment effectively inhibited iNOS, ICAM-1 and TNF-α activity in the lung after ANP. However, the expression of IL-10 was up-regulated in lung pretreatment with TDZD-8 or SB216763. TDZD-8 or SB216763 pretreatment induced a signiﬁcant increase in phospho-GSK-3β (Ser9). The expression of phospho-GSK-3β (ser9) in the ANP-TDZD-8 group was slightly stronger than that in the ANP-SB216763 group. Comparing the ANP-TDZD-8 group with the ANP-SB216763 group, it was found that except for TNF-α, the expression of iNOS, ICAM-1 in the ANP-TDZD-8 group was lower than that in the ANP-SB216763 group. B, C, D, E. Western blots were quantitated for iNOS, ICAM-1, IL-10 and TNF-α to β-action. F. Western blots were quantitated for phospho-GSK-3β (Ser9) to GSK-3β. The results are expressed as a fold-increase over sham-vehicle in at least 3 independent experiments. Results are presented as the mean ± standard deviation. Statistical significance was determined using Student’s two-tailed t-test. *P < 0.05, **P < 0.01, ***P < 0.001. ****P < 0.0001.

To obtain the mechanism of action of the GSK-3β inhibitors, Western blot analysis of p-GSK-3β (Ser9) was performed. From Figure 3A, p-GSK-3β (ser9) in lung tissue had a remarkable increase in the ANP-TDZD-8 and ANP-SB216763 groups over ANP-vehicle group (P<0.01). Moreover, the expression of p-GSK-3β (ser9) in ANP-TDZD-8 group was slightly stronger than that in the ANP-SB216763 group (P<0.01) (Fig. 3F).

### GSK-3β inhibitors attenuate activation of iNOS, ICAM-1 and TNF-α in lung tissues after ANP

In a word, all of such findings revealed the gradual aggravation in the degree of pancreatitis and its associated lung injuries. Consequently, we determined the efficacy of TDZD-8 or SB216763 on iNOS, ICAM-1 and TNF-α expression in the lung after 12 hours. As shown in Figure 3 A-E, expression of iNOS, ICAM-1 and TNF-α in lung tissue was significantly elevated in the ANP-vehicle group versus the sham group (*P*<0.05), but inhibitory function of GSK-3β remarkably abated the activation of iNOS, ICAM-1 and TNF-α in the lung in ANP (*P*<0.05). Moreover, comparing the ANP-TDZD-8 group with the ANP-SB216763 group, it was found that except for TNF-α, the expression of iNOS, ICAM-1 in the ANP-TDZD-8 group was lower than that in the ANP-SB216763 group (*P*<0.05).

### GSK-3β inhibitors up-regulate activation of IL-10 in lung tissues after ANP

According to the western blot analysis result in Figure 3D, the expression of IL-10 decreased significantly in the lung following ANP. However, pretreatment with TDZD-8 or SB216763 increased the activation of IL-10 in lung tissues (*P*<0.01). In addition, the expression of IL-10 in the ANP-TDZD-8 group was higher than that in the group ANP-SB216763 group (*P*<0.05).

## Discussion

The current research made a preliminary exploration on the effect of different GSK-3β inhibitors on ANP-related lung injuries and its latent mechanism. These outcomes demonstrated a great protection effect of GSK-3β inhibitors against acute lung injuries in ANP. In addition, two different GSK-3β inhibitors are capable of reducing serum amylase and lipase concentrations, pro-inflammatory mediators, pancreatic damage and lung injury. It can also be demonstrated that the protective effect of GSK-3β inhibitors on ANP and related lung injury is achieved by inactivating the NF-κB signaling pathway. All the findings present that GSK-3β inhibitors have an underlying anti-inﬂammation effect and improve the degree of ANP and related lung injuries in rats. At the same time, we also found that the effects of different GSK-3β inhibitors are different, and TDZD-8 shows better anti-inflammatory effects than SB216763. Inhibiting GSK-3β is deemed to suppress pro-inflammatory in several inflammatory diseases including lung injury^12,14^.Could GSK-3β inhibitors protect the lungs from inflammatory damage caused by ANP? We found that GSK-3β inhibitors reduced the expression of NF-κB p65 in lung tissue of ANP rats. A variety of treatment approaches targeted at the NF-κB signaling pathway like antioxidants, anti-inflammatory agents, or pharmacologic inhibition revealed effectiveness in experimental AP models. NF-κB needs to be transferred from the cytoplasm to the nucleus to activate the NF-κB pathway, which binds to the promoter regions of various pro-inflammatory genes and activates transcription^18^. Cumulative evidence shows that NF-κB p65, especially the COOH-terminus of p65 at Ser 536, can be phosphorylated by GSK-3β, to strengthen transcriptional responses of NF-κB^19^. The peculiarity of GSK-3β rests with its capability of affecting the activity of the transcription factor NF-κB^20^. Some studies found that GSK-3β knockout rats showed an identical phenotype to that of rats that underwent the deletion of the gene for NF-κB p65^21^. Hence, pharmacologic inhibition of GSK-3β is likely to cause inhibition of the NF-κB transcriptional activity. Our immunohistochemical analysis revealed NF-κB is activated in lung tissue and transferred from cytoplasm to nucleus and a signiﬁcant inhibition of NF-κB p65 by GSK-3β inhibitors in the lung tissues of rats, which conﬁrms GSK-3β inhibitors could suppress activation of NF-κB signaling pathway in lung after ANP.

Inhibitors of GSK-3β are evident to decrease the activation of NF-κB, which in turn will cause a decreased formation of pro-inflammatory cytokines (e.g., IL-1β, IL-6, and TNF-α)^22^. Cytokines such as IL-1β, IL-6 and TNF-α help to propagate the extension of a local or systemic inflammatory process containing ANP. Our data revealed a growth in the cytokines after ANP. Nevertheless, the serum level of IL-1β and IL-6 was significantly lower in rats treated with GSK-3β inhibitors, and TNF-α expression in lung tissues also decreased. Such observations converge with preceding reports revealing a significant reduction of IL-1β and IL-6 levels in serum of rats subjected to AP by cerulein through chemically distinct GSK-3β inhibitors^14^. Various studies have clearly reported that TNF-α is deemed to be a key cytokine initiator for the inﬂammatory cascade of AP and the degree of pancreatic injuries in AP has a direct correlation with the level of TNF-α^23^. Application of anti-TNF-α antibody or TNF-α antagonist etanercept in animal AP model can significantly reduce serum amylase and lipase, relieve the degree of pancreas and associate-lung injury, and improve animal survival rate^24^. TNF-α inhibitors obviously alleviate the progression of the inflammation procedure in AP patients^25^. Hence, it is proposed that the inhibitory function of IL-1β, IL-6 and TNF-α formation in GSK-3β inhibitors-treated rats found in current research could be attributed to a GSK-3β-mediated inhibition of the activation of NF-κB.

Strengthened formation of nitric oxide by iNOS is likely to cause the inflammatory process related to acute pancreatitis^23^. Those outcomes revealed the increase in the expression level of iNOS in lung tissue after induction of ANP. It intensively suggests the involvement of iNOS in the progression of ANP-related acute lung injuries. However, this study demonstrated that GSK-3β inhibitors alleviate expression of iNOS in the lung tissues of ANP rats. It could be supposed that the low level expression of iNOS-generated proper level nitric oxide in normal physiological conditions was good to the body. However, at the induction of ANP, the excessive nitric oxide might lead to microvascular endothelium cells injuries and dysfunctions, causing growth in vascular permeability; on the other hand, over-produced nitric oxide might produce oxygen-free radical to enhance cytotoxic effects, exacerbating lung injury^26^. Such decrease in the expression of iNOS by GSK-3β inhibitors is likely to cause the abatement of nitrotyrosine formation and lipid peroxidation in the lung in ANP animals. These results suggest that GSK-3β pathway inhibition abates iNOS expression.

As a key adhesion molecule, ICAM-1 plays an important role in the pathogenesis of ANP. ICAM-1 can mediate leukocyte adhesion and migration, allowing white blood cells to be recruited in the pancreas and distant organs, leading to pancreas and distant organs injury in acute pancreatitis^16^. In line with our observation, ANP causes an apparently elevated expression of ICAM-1 in lung tissues. Treatment with GSK-3β inhibitors down-regulated the expression of ICAM-1 in lung tissue. Such outcomes reveal the interference of inhibition of the GSK-3β pathway to the neutrophil recruitment as well as its support to the concept of a treatment approach directed against leukocyte infiltration in lung injury.

IL-10 is secreted primarily by helper T-lymphocytes and mononuclear macrophages, and has an anti-inflammatory effects through T cells^27^. IL-10 restores the balance between the anti-inflammatory response and the inflammatory response via inhibition of the body’s inflammatory response^28^. Our research observes that the level of IL-10 in lung is reduced in STC-induced ANP rats, so it is insufficient to have an anti-inflammatory effect, aggravating inflammatory response in the lung. However, we found that the expression of IL-10 was up-regulated in lung when we used GSK-3β inhibitors. Therefore, there is a severe imbalance between the anti-inflammatory response and the inflammatory response in rats with ANP, whereas GSK-3β inhibitors are able to maintain the balance between the anti-inflammatory response and the inflammatory response through inhibitory expression levels of inflammation factors and promoted expression of anti-inflammatory factor IL-10 in lung, thus abating lung injuries.

The GSK-3β inhibitors currently used are quite different in chemical structure. They are mainly classified into small molecule ATP competitive inhibitors and non-ATP competitive inhibitors. Small molecule ATP competitive inhibitors are mostly small molecule hydrophobic heterocyclic compounds with a relative molecular mass of generally less than 600. Most of them inhibit the GSK-3β activity by competitively binding to ATP on the GSK-3β Ser9 phosphorylation site. SB216763 is one of the representative products of molecular ATP competitive inhibitors. Non-ATP competitive inhibitors are generally classified by the inorganic ion inhibitors lithium (Li) and TDZD-8. Li is the first GSK-3 inhibitor found but the mechanism by which lithium inhibits GSK-3 activity is not fully understood. TDZD-8 is a non-ATP competitive small molecule inhibitor of GSK-3β, which mainly phosphorylates GSK-3β ser9 to reduce the activity of GSK-3β. It is characterized by not blocking the activity of other types of kinases such as protein kinases A and C, casein kinase II and cyclin-dependent kinase-1^29^. However, some studies have found that TDZD-8 can also act as a small molecule inhibitor of ATP competition, because its binding site can compete with ATP for the catalytic region in addition to the T-loop region^30^. Therefore, it can be considered that TDZD-8 has a dual inhibitory effect. We compared the effects of two different GSK-3β inhibitors, TDZD-8 and SB216763, on ANP rats. Both inhibitors have protective effects on lung injury in ANP rats, reducing serum amylase and lipase values and can reduce the pathological scores of pancreas and lungs. However, we compared the effects of TDZD-8 and SB216763 on serum amylase, lipase, pancreas and lung pathology scores, and found that TDZD-8 has a better protective effect on ANP lung injury than SB216763. At the same time, we found that IL-1β, IL-6 in serum and iNOS, ICAM-1, TNF-a expression in Western, ANP-TDZD group were also lower than ANP-SB216763, but the expression of p-GSK-3β Ser9 is higher. This may be due to the fact that TDZD-8 has a dual inhibitory effect of non-ATP competitive small molecule inhibition and ATP competitive inhibition.

## Conclusions

This study demonstrates the mechanism by which GSK-3β inhibitors alleviate lung injuries depends in part on attenuating the activation of the NF-κB pathway, resulting in a decreased expression of pro-inflammatory cytokines. At the same time, the expression of anti-inflammatory factor was up-regulated. In addition, TDZD-8 has better protection against lung injury than SB216763 related to TDZD-8 has a dual inhibitory effect of non-ATP competitive small molecule inhibition and ATP competitive inhibition. The observations presented in current research are likely to arise more attention to the progression of GSK-3β inhibitors with greater specificity that can be utilized alone or jointly to prevent and treat ANP as well as related lung injuries.

## Footnotes

1 Research performed at the Department of Hepatobiliary Surgery, Renmin Hospital, Wuhan University, Hubei, China.
